# miR-135 family members mediate podocyte injury through the activation of Wnt/β-catenin signaling

**DOI:** 10.3892/ijmm.2015.2259

**Published:** 2015-06-24

**Authors:** XIANGGUI YANG, XIAOYAN WANG, FANG NIE, TIANMING LIU, XUEJING YU, HONGLIAN WANG, QIANYIN LI, RUI PENG, ZHAOMIN MAO, QIN ZHOU, GE LI

**Affiliations:** 1 Division of Molecular Nephrology and Creative Training Center for Undergraduates, The M.O.E. Key Laboratory of Laboratory Medical Diagnostics, College of Laboratory Medicine, Chongqing Medical University; Chongqing, P.R. China; 2 The Core Facility of Experiments Training, Chongqing Medical University; Chongqing, P.R. China; 3 Department of Cardiology, The Fifth Medical College, Peking University, Beijing, P.R. China; 4 Research Center of Combined Traditional Chinese and Western Medicine, The Affiliated Traditional Medicine Hospital, Luzhou Medical College, Luzhou, Sichuan, P.R. China

**Keywords:** miR-135a, miR-135b, podocytes, injury, wnt/β-catenin signaling, glycogen synthase kinase 3β

## Abstract

The upregulation of Wnt/β-catenin signaling occurs in virtually all types of kidney disease and is associated with podocyte injury. However, the precise mechanisms involved in the development of kidney disease remain to be elucidated. MicroRNAs (miRNAs or miRs) are a class of short non-coding RNAs and they have been shown to be regulators of gene expression, mainly by binding to the untranslated region (UTR) of mRNAs. The aim of the present study was to determine the role of the 2 members of the miR-135 family (miR-135a and miR-135b) in podocyte injury and to elucidate the mechanisms responsible for the damage to podocytes. The results revealed that miR-135a and miR-135b were upregulated in models of podocyte injury and in glomeruli isolated from patients with focal segmental glomerulosclerosis (FSGS). The ectopic expression of miR-135a and miR-135b led to severe podocyte injury and the disorder of the podocyte cytoskeleton. Our findings demonstrated that miR-135a and miR-135b activated Wnt/β-catenin signaling and induced the nuclear translocation of β-catenin. Using luciferase reporter assays, reverse transcription-quantitative polymerase chain reaction (RT-qPCR) and western blot analysis, glycogen synthase kinase 3β (GSK3β) was identified as a target gene of miR-135a and miR-135b. To the best of our knowledge, this is the first study to demonstrate that members of the miR-135 family (specifically miR-135a and miR-135b) regulate the expression of GSK3β, thus playing a role in the development of podocyte injury and the disorder of the podocyte cytoskeleton. This is an important finding as it may contribute to the development of novel therapeutics for podocyte injury-associated glomerulopathies.

## Introduction

A large number of secreted glycoproteins involved in organogenesis, stem cell homeostasis and tumor formation encoded by the Wnt gene have been verified from the *Hydra* to the human genome (1). The enhanced activation of Wnt/β-catenin signaling and β-catenin nuclear translocation have been shown to play a role in podocyte injury *in vivo* and *in vitro* (3). The administration of puromycin to cultured podocytes has been demonstrated to induce the nuclear translocation of β-catenin (2). The Wnt/β-catenin signaling pathway may also be regulated by transforming growth factor-β (TGF-β) and adriamycin (ADR) *in vitro* (3,4). Increased podocyte Wnt/β-catenin signaling has also been observed in podocytes in murine models of diabetic nephropathy and focal segmental glomerulosclerosis (FSGS) (3,5). These results indicate a high consistency in the activation of Wnt/β-catenin signaling in podocytes in response to various types of injury and various diseases. However, the potential mechanisms involved remain poorly understood.

MicroRNAs (miRNAs or miRs) are a class of small non-coding RNAs which play indispensible roles in the regulation of gene expression through translational repression or transcript degradation (6). Recently, studies have indicated that miRNAs play a key role in kidney diseases. miR-93 has been shown to facilitate glomerular injury through the activation of vascular endothelial growth factor (7). By targeting Bcl-2, miR-195 aggravates podocyte apoptosis (8). The downregulation of miR-30 has also been shown to promote podocyte injury (9). Studies have demonstrated that miR-192 accelerates collagen formation in glomerular mesangial cells in models of diabetic nephropathy (10) and promotes TGF-β/Smad3-induced tubulointerstitial fibrosis (11). The loss of Dicer in podocytes has been shown to lead to the development of proteinuria and glomerulosclerosis (12). These studies indicate that miRNAs play indispensable roles in the development of glomerular diseases, particularly podocyte-associated disorders. However, the underlying mechanisms have not yet been fully delineated.

The miR-135 family is highly conserved among mammals and consists of 2 members, miR-135a and miR-135b. It has been reported that miR-135a and miR-135b function as oncogenes and play prominent roles in the development of various types of cancer, including the pathogenesis of colorectal cancer (13), a role in the promotion of paclitaxel resistance in non-small cell lung cancer (14) and in the facilitation of growth and invasion in colorectal cancer (15). However, other studies have demonstrated that miR-135a is a tumor suppressor gene that inhibits cell proliferation in renal cancer (16) and selectively kills malignant glioma cells (17). Additionally, miR-135a determines the size of the midbrain during its development (18) and promotes renal fibrosis in diabetic nephropathy (19). Despite these findings, the exact function of the two miR-135 family members remains largely unknown, particularly their function in podocyte injury-associated renal diseases.

In the present study, we aimed to determine the roles and mechanisms of action of miR-135a and miR-135b in podocyte injury, and to elucidate the mechanisms underlying podocyte injury. We found that miR-135a and miR-135b were overexpressed in patients with FSGS and in models of podocyte injury, and that the ectopic expression of these miRNAs promoted podocyte injury by activating Wnt/β-catenin signaling through the suppression of glycogen synthase kinase 3β (GSK3β) expression. Our findings demonstrate that miR-135a and miR-135b play an important role in podocyte injury. Our findings may provide new insight into the understanding of the molecular mechanisms underlying podocyte injury, which may be crucial for the development of novel therapeutic agents for the treatment of podocytopathy.

## Materials and methods

### Ethics statement

Approval for human subject research in the present study was granted by the Ethics Committee of the First Affiliated Hospital of Chongqing Medical University, Chongqing, China. Each patient enrolled in this study provided written informed consent. For the animal experiments, all surgical procedures performed on mice were carried out under sodium pentobarbital anesthesia (5 mg/100 g). The animals were treated according to the recommendations listed in the Guide for the Care and Use of Laboratory Animals of the National Institutes of Health (NIH). The experimental procedures were approved by the Animal Experimental Ethics Committee of Chongqing Medical University.

### Animal experiments

BALB/c mice (6–7 weeks old) were purchased from the Animal Center Management, Chongqing Medical University. The mouse models of podocyte injury were created by the administration of an intravenous injection of ADR (10.5 mg/kg; Sigma, St. Louis, MO, USA), as previously described (20,21). The mice in the control group received an equivalent volume of normal saline (NS). The mice were sacrificed by cervical vertebra dislocation on days 4, 7, 11, 15 and 20 following the administration of ADR or NS. The glomeruli were isolated from the kidneys using a sieving technique as previously described (22). Mice were anesthetized and the kidneys were removed, minced into 1 mm^3^ sections and digested in collagenase (1 mg/ml collagenase A, 100 U/ml deoxyribonuclease I in HBSS) at 37°C for 30 min with gentle agitation. The collagenase-digested tissue was gently pressed through a 100-*µ*m cell strainer using a flattened pestle and the cell strainer was then washed with 5 ml of HBSS. The filtered cells were passed through a new cell strainer without pressing and the cell strainer washed with 5 ml of HBSS. The cell suspension was then centrifuged at 200 × g for 5 min. The supernatant was discarded and the cell pellet was resuspended in 2 ml of HBSS. Finally, glomeruli were gathered by centrifugation (at 200 × g for 5 min). They were then stored in liquid nitrogen until further analysis.

### Human samples

Three patients diagnosed as having FSGS by a renal biopsy at the Department of Nephrology, the First Affiliated Hospital, Chongqing Medical University were enrolled in the present study. The control group included 3 patients with kidney rupture following incidents of violence or traffic accidents. The glomeruli were isolated from the renal tissues using the aforementioned sieving technique and were then snap-frozen in liquid nitrogen for RNA extraction and molecular analysis. Podocyte damage was determined by analysis of the pathological sections and albuminuria (27).

### Cell culture and treatment

293T cells were cultured in Dulbecco's modified Eagle's medium (DMEM) supplemented with 10% fetal bovine serum (Gibco-BRL, Carlsbad, CA, USA) at 37°C with 5% CO_2_. The conditionally immortalized mouse podocyte cell line 5 (MPC5) was cultured as previously described (23). MPC-5 cells were cultured at 33°C (10 U/ml of interferon-γ) with 5% CO_2_ and were differentiated at 37°C (without interferon-γ) with 5% CO_2_ in RPMI Dutch-modified medium (Invitrogen, Carlsbad, CA, USA) supplemented with 10% v/v fetal calf serum. Depending on the exact experimental setup, the cultured cells were treated with different doses of adriamycin and transfected with microRNA mimics or plasmid using Lipofectamine 3000 reagent (Invitrogen, Grand Island, NY, USA). NT group cells were treated without any agents.

### Bioinformatics analysis

The miR-135 candidate genes were predicted using the following websites: miRwalk (www.umm.uni-heidelberg.de/apps/zmf/mirwalk), miRanda (www.microrna.org/microrna/home.do) and TargetScan (www.targetscan.org/).

### Constructs and luciferase assays

The 3′ untranslated region (3′UTR) of GSK3β was obtained by the amplification of genomic DNA using a forward primer (5′-GCTCCTCACCAAATTCAAGC-3′) and a reverse primer (5′-TGAAGTGAGACTGCCAACATG-3′). The PCR product was cloned into the pcDNA3.1-Luc reporter vector and verified by sequencing. For the wild type construct, the seed sequence was wild type. For the mutant construct, the seed sequence was mutated. miR-135 inhibitor (Inh-135) was from RiboBio Co., Ltd., Guangzhou, China. The microRNA control inhibitor (Inh-NC) was from RiboBio Co., Ltd. Luciferase assays were conducted according to a method described in a previous study (24). In brief, the 293T and MPC5 cells were cultured in 24-well plates and transfected with 500 ng of the repoter vector, 50 ng of pRL-CMV and 50 nM of miR-135a/miR-135b mimics or the miRNA mimic negative control (RiboBio Co., Ltd.) using Lipofectamine 3000 reagent (Invitrogen, Grand Island, NY, USA). The firefly and *Renilla* luciferase activities were measured at 36 h after transfection using a dual-luciferase assay system (Promega, Madison, WI, USA) according to the manufacturer's instructions. Dishevelled regulates GSK3β activity. This results in the stabilization and accumulation of cytosolic β-catenin, a GSK-3β substrate, which is when phosphorylated by GSK-3β, and is targeted for ubiquitin-mediated proteolysis. β-catenin then translocates to the nucleus, where in complexes with members of the TCF family of transcription regulators, activates the transcription of TCF-responsive genes. This TCF-reporter plasmid enables the quantification of Wnt signaling in cells transfected with these constructs. LiCl can repress GSK-3β expression, this results in the stabilization and accumulation of cytosolic β-catenin.

### RNA extraction and reverse transcription-quantitative PCR (RT-qPCR)

RNA was extracted using TRIzol reagent (Ambion, Austin, TX, USA) according to the manufacturer's instructions. RT-qPCR for the detection of miR-135a and miR-135b was performed using miR-135a- and miR-135b-specific PCR primers (RiboBio Co., Ltd.) with the RevertAid First Strand cDNA Synthesis kit (Fermentas, Burlington, ON, Canada) and SYBR Premix Ex Taq™ II (Takara, Dalian, China) according to the manufacturers' instructions. For the mRNA quantification of protein-encoding genes, the RNA was reverse transcribed with a random primer and the mRNA levels were determined by RT-qPCR. All the sequences of the primers using in RT-qPCR primers are presented in Table I.

### Protein extraction and western blot analysis

Total protein was extracted from the MPC5 cells using RIPA lysis buffer (Beyotime, Jiangsu, China) following the manufacturer's instructions. Western blot analysis was performed as previously described (25). The antibodies used were the following: rabbit anti-desmin (1:1,000; sc-14026), rabbit anti-snail (1:1,000; sc-28199) (all from Santa Cruz Biotechnology, Inc., Santa Cruz, CA, USA), rabbit anti-nephrin (1:2,000; ab136894; Abcam, Cambridge, MA, USA), rabbit anti-Wilms tumor 1 (WT1; 1:500; sc-192), rabbit anti-E-cadherin (1:1,000l; sc-7870) (both from Santa Cruz Biotechnology, Inc.), rabbit anti-GSK3β (1:2,000; ab18893; Abcam), rabbit anti-β-catenin [activated and unphosphorylated, 1:2,000; Netherlands Cancer Institute (NKI), Amsterdam, The Netherlands], mouse anti-β-tubulin (1:3,000; sc-80011), goat anti-rabbit IgG-HRP (1:3,000; sc-2004) and goat anti-mouse IgG-HRP (1:3,000; sc-2005) (all from Santa Cruz Biotechnology, Inc.). For the quantitative analysis of the western blots, the protein band intensities were quantified using Image J software and β-actin was used for normalization.

### F-actin cytoskeleton staining and immunofluorescence staining

F-actin was stained using rhodamine-labeled phalloidin (diluted in PBS containing 5% BSA, 1:1,000; Sigma) according to the manufacturer's instructions. Immunostaining for the determination of the location of β-catenin was performed as previously described (26). The following antibodies were used: rabbit anti-β-catenin (1:1,000; ab32572; Abcam) and goat anti-rabbit IgG-CFL 488 (1:2,000; sc-362262; Santa Cruz Biotechnology, Inc.).

### Statistical analysis

All the results are presented as the means ± SD. The t-test was employed to determine whether 2 sets of data (groups) differed significantly from each other using GraphPad Prism 5 software (GraphPad Software, Inc., La Jolla, CA, USA). Values of P<0.05 and P<0.01 were considered to indicate statistically significant and highly statistically significant differences, respectively. All experiments were performed at least 3 times.

## Results

### miR-135a and miR-135b are upregulated in injured podocytes

**Figure 1:**
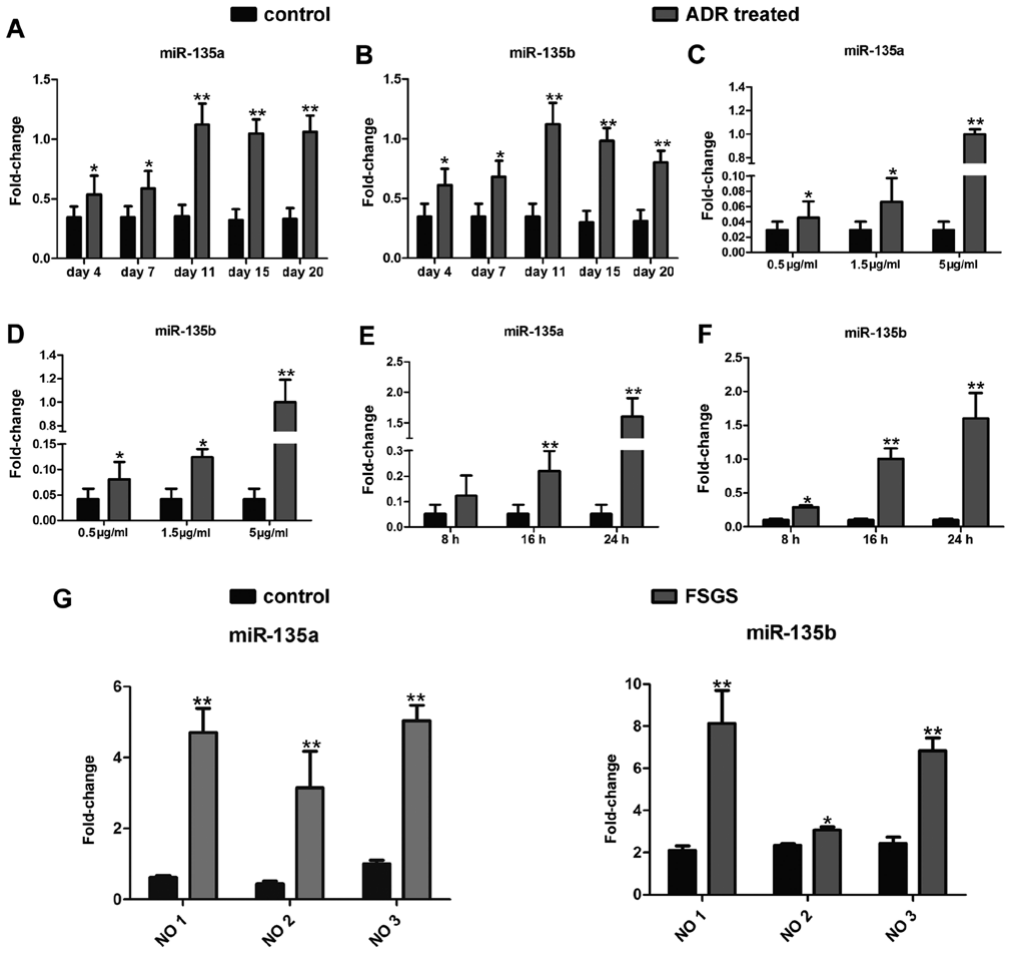
miR-135a and miR-135b are upregulated in injured podocytes. (A and B) Quantification by RT-qPCR of (A) miR-135a and (B) miR-135b expression in the isolated glomeruli from BALB/c mice treated with adriamycin (ADR; 10.5 mg/kg) at different time points after the injection (n=3, means ± SD). (C–F) miR-135a and miR-135b expression levels were measured in the cultured mouse podocyte cell line 5 (MPC5) treated with ADR (C and D) at different doses and (E and F) for different periods of time. (G) Quantification by RT-qPCR of miR-135a and miR-135b in the glomeruli isolated from 3 patients with focal segmental glomerulosclerosis (FSGS) and 3 patients with kidney rupture (controls). ^*^P<0.05 and ^**^P<0.01 indicacte statistically significant and highly statistically significant differences, respectively, compared with the controls. NO1, number 1 patient; NO2, number 2 patient; NO3, number 3 patient.

To investigate the role of miR-135a and miR-135b in podocyte injury, firstly, a mouse model of ADR-induced nephropathy, a widely recognized model of podocyte injury (20,21), was established by an intravenous injection of ADR (10.5 mg/kg). The expression levels of both members of the miR-135 family were detected in the isolated glomeruli by RT-qPCR as indicated in Fig. 1A and B. The expression levels of miR-135a and miR-135b increased significantly as early as day 4 following the administration of ADR. Eleven days following the administration of ADR, the miR-135a and miR-135b expression levels increased by approximately 3-fold. Furthermore, the expression levels of both miRNAs were measured in an *in vitro* model of ADR-induced podocyte injury. As demonstrated in Fig. 1C–F, treatment with ADR enhanced the expression of miR-135a and miR-135b in the cultured mouse podocyte cell line (MPC5) in a dose-dependent (Fig. 1C and D; 24 h after ADR treatment) and time-dependent manner (Fig. 1E and F; 5 *µ*g/ml ADR treatment). Additionally, in order to further investigate the clinical significance of miR-135a and miR-135b in podocyte injury, glomeruli were isolated from 3 patients with FSGS whose podocytes were severely damaged and from 3 patients with kidney rupture caused by incidents of violence or traffic accidents. The renal samples from patients with kidney rupture were used as controls. miR-135a and miR-135b expression in the glomeruli from these patients was examined by RT-qPCR. As expected, miR-135a and miR-135b were highly expressed in the glomeruli of the patients with FSGS compared with those of the controls (Fig. 1G). Thus, the aforementioned results clearly indicate that miR-135a and miR-135b are upregulated in injured podocytes.

### Overexpression of miR-135 family members leads to podocyte injury

**Figure 2:**
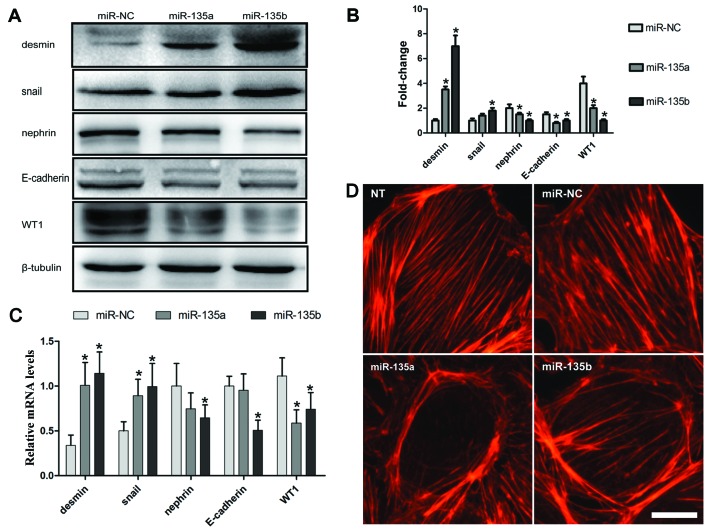
miR-135a and miR-135b promote podocyte damage and cytoskeletal injury. (A) Western blot analysis was applied to determine the changes in the expression levels of desmin, snail, nephrin, E-cadherin and WT1 at 48 h following transfection of the cultured mouse podocyte cell line 5 (MPC5) with miR-135a and miR-135b mimics of the negative control (miR-NC). (B) Quantitative analysis of the results of western blot analysis. The intensities of the protein bands were quantified and normalized to β-tubulin. (C) Quantification by RT-qPCR of the mRNA expression of desmin, E-cadherin, nephrin, WT1 and snail following transfection of the MPC5 cells with miR-135a and miR-135b mimics or miR-NC. (D) Effects of miR-135a and miR-135b on cytoskeletal stabilization in podocytes. Scale bar, 10 *µ*m. NT, not treated. ^*^P<0.05 and ^**^P<0.01 indicate statistically significant and highly statistically significant differences, respectively, compared with the miR-NC group.

To determine whether the increase in the expression of miR-135a and miR-135b is functionally significant in podocyte injury, miR-135a and miR-135b mimics and miRNA mimic negative control (miR-NC) were transfected into the MPC5 cells. Forty-eight hours after transfection, the protein expression levels of markers related to podocyte injury were measured by western blot analysis. The expression of desmin and snail, two markers of podocyte injury (3,28), significantly increased in the cells transfected with miR-135a and miR-135b mimics compared with the miR-NC group (Fig. 2A and B). However, the expression of the podocyte functional markers, WT1, nephrin and E-cadherin, was suppressed by transfection with miR-135a and miR-135b mimics (Fig. 2A and B). Furthermore, the mRNA expression of these genes was determined by RT-qPCR, the results of which were consistent with those of the protein expression (Fig. 2C).

Any factor that can cause the disorder or rearrangement of the podocyte cytoskeleton may result in podocyte dysfunction and injury (29). Therefore, in this study, the effects of miR-135a and miR-135b on the podocyte cytoskeleton were examined. The podocyte cytoskeleton was examined by means of fluorescein-conjugated phalloidin staining at 36 h following transfection with miR-135a and miR-135b mimics or miR-NC. The results revealed that miR-135a and miR-135b promoted the rearrangement of stress fibers in podocytes, causing them to localize around the cytomembrane compared with the control-transfected and untreated groups (NT) (Fig. 2D).

### miR-135a and miR-135b promote Wnt/β-catenin signaling in MPC5 cells

**Figure 3:**
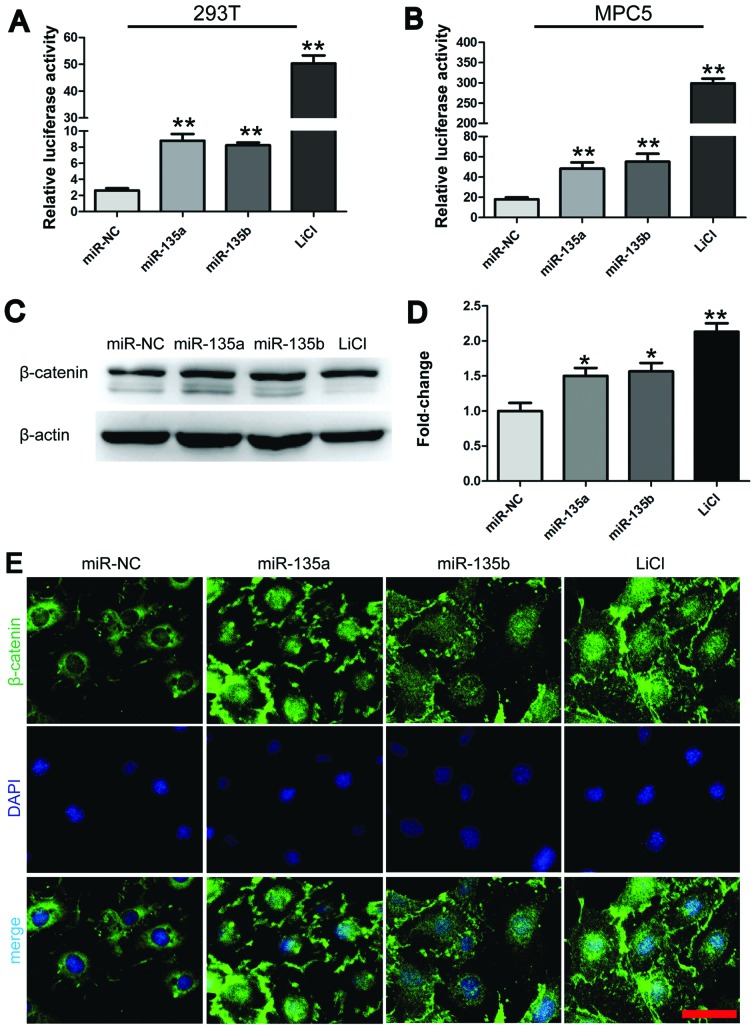
miR-135a and miR-135b promote the activation of wnt/β-catenin signaling. (A and B) Dual-luciferase assays demonstrating the effects of miR-135a and miR-135b on TOPflash reporter activity in (A) 293T cells and (B) in the mouse podocyte cell line 5 (MPC5). (C) Western blot analysis of the changes in the expression of activated and unphosphorylated β-catenin in cultured MPC5 cells transfected with miR-135a and miR-135b mimics or negative control miR-NC. (D) Quantitative analysis of the results of western blot analysis. The protein band intensities of β-catenin were quantified using ImageJ software and normalized to β-actin. (E) Utilization of immunostaining to identify the effects of miR-135a and miR-135b on the subcellular localization of β-catenin. LiCl (10 mM) was used as a positive control in these experiments. Scale bar, 50 *µ*m. ^*^P<0.05 and ^**^P<0.01 indicate statistically significant and highly statistically significant differences, respectively, compared with the miR-NC group.

As is well known, desmin and snail are potential downstream targets of the Wnt/β-catenin signaling pathway (30). As shown in Fig. 2A, desmin and snail were overexpressed in the injured podocytes. Therefore, we hypothesized that miR-135a and miR-135b mediate podocyte injury through Wnt/β-catenin signaling. To verify this hypothesis, we first examined the effects of miR-135a and miR-135b on T-cell factor (TCF) transcriptional activity. The 293T and MPC5 cells were co-transfected with miR-135a and miR-135b mimics or miR-NC and luciferase reporter constructs harboring 3 optimal TCF binding sites (TOPflash). The lithium chloride (LiCl; 10 mM) group was used as a positive control. The results revealed that transfection with either miR-135a or miR-135b significantly increased TOPflash reporter activity by approximately 3-fold compared with the negative control (Fig. 3A and B). Subsequently, the effects of miR-135a and miR-135b on the expression levels of activated and unphosphorylated β-catenin were examined in the MPC5 cells by western blot analysis. In accordance with previous findings (13), we found that the overexpression of miR-135a and miR-135b increased the levels of activated and unphosphorylated β-catenin by approximately 1.5-fold compared with the negative control (Fig. 3C and D). This effect was very similar to that observed in the group treated with LiCl (10 mM). Furthermore, immunofluorescence staining for β-catenin was conducted to examine the effects of miR-135a and miR-135b on the subcellular location of β-catenin in the MPC5 cells. The nuclear translocation of β-catenin occurred following transfection of the cells with miR-135a and miR-135b mimics and was comparable to that in the group treated with LiCl (10 mM) (Fig. 3E). By contrast, β-catenin was predominantly localized at the cell-cell adhesion sites and around the nucleus in the MPC5 cells in the control group (Fig. 3E). Thus, the aforementioned findings indicate that miR-135a and miR-135b activate Wnt/β-catenin signaling.

### miR-135a and miR-135b target the 3′UTR of GSK3β and inhibit GSK3β expression

**Figure 4:**
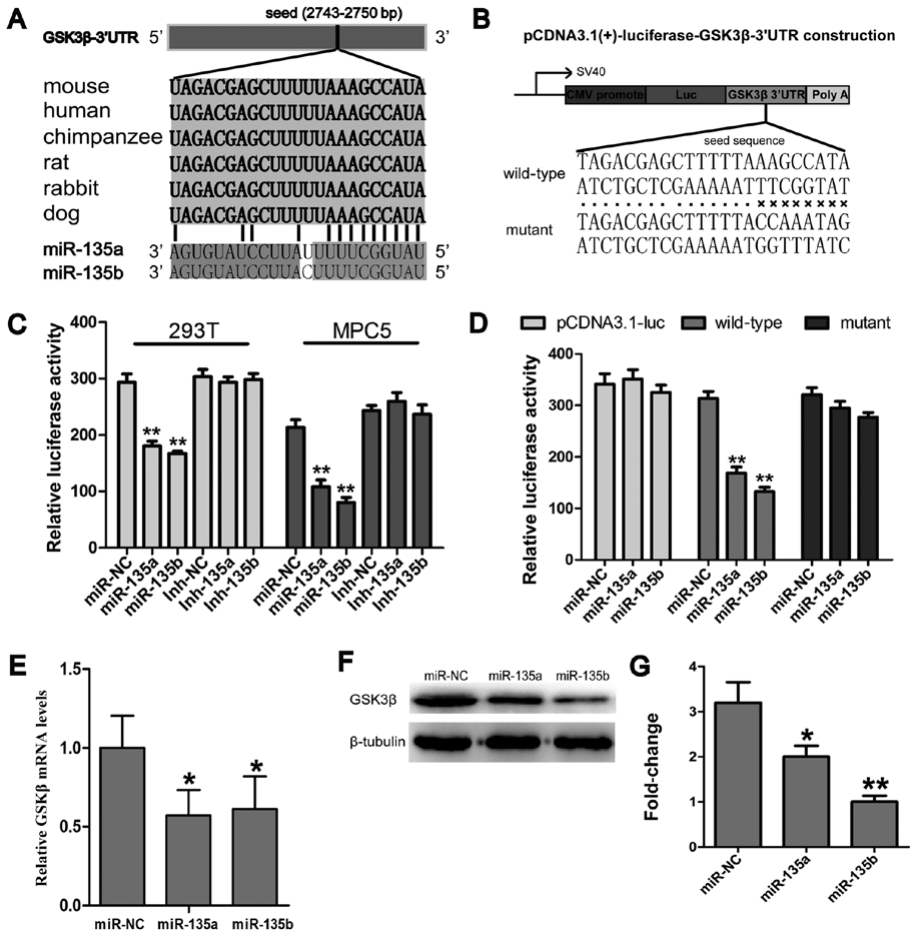
Glycogen synthase kinase 3β (GSK3β) is a target gene of miR-135a and m iR-135b. (A) The predicted binding sites of miR-135a and miR-135b in the 3′ untranslated region (3′UTR) of GSK3β mRNA, the sequence alignment between miR-135a and miR-135b and GSK3β 3′UTR and conservational analysis of GSK3β 3′UTR among different mammals. (B) Schematic diagram of GSK3β 3′UTR reporter constructs, including wild-type and mutant vectors. (C and D) Dual luciferase reporter assays. (C) 293T cells and the mouse podocyte cell line 5 (MPC5) were transfected with pCDNA3.1-luciferase-GSK3β-3′UTR reporter and miR-135s/Inh-135s/miR-NC/Inh-NC. (D) One of the pCDNA3.1-luciferase-GSK3β-3′UTR reporter, pCDNA3.1-luciferase-GSK3β-3′UTR mutant reporter and pCDNA3.1-luciferase reporter was co-transfected into the MPC5 cells with miR-135a and miR-135b or miR-NC. (E) RT-qPCR and (F) western blot analysis for measuring GSK3β expression following transfection with miR-135a and miR-135 mimics or miR-NC. (G) Quantitative analysis of GSK3β protein expression. Inh-135s, miR-135a and miR-135b inhibitor. Inh-NC, negative control of miRNA inhibitor. ^*^P<0.05 and ^**^P<0.01 indicate statistically significant and highly statistically significant differences, respectively, compared with the miR-NC group.

In order to further elucidate the molecular mechanisms underlying the miR-135a- and miR-135b-induced activation of Wnt/β-catenin signaling, the targets of miR-135a and miR-135b involved in Wnt/β-catenin signaling were identified by online miRNA target prediction programmes, including miRwalk, miRanda and TargetScan release 6.2. The analysis revealed that an evolutionarily conserved region in the 3′UTR of GSK3β mRNA had a perfect complementary sequence (5′-UGUUUACA-3′) to the seed sequence (3′-ACAAAUGU-5′) of the miR-135 family (Fig. 4A). In order ro further validate the results of bioinformatics analysis, reporter vectors bearing the GSK-3β 3′UTR were constructed (Fig. 4B). Luciferase reporter assays were conducted to determine whether miR-135a and miR-135b directly target the 3′UTR of GSK3β. The results revealed that luciferase expression was significantly suppressed in the groups transfected with the miR-135a and miR-135b mimics compared with the miR-NC group, while mutations of the miR-135a and miR-135b binding sites abrogated the suppression of luciferase expression (Fig. 4C and D), which suggests that miR-135a and miR-135b directly bind to the 3′UTR of GSK3β. Furthermore, the effects of miR-135a and miR-135b on GSK3β expression at the protein and mRNA level were also determined. The results revealed that miR-135a and miR-135b inhibited the expression of GSK3β at both the protein and mRNA level (Fig. 4E–G). Taken together, these results indicate that both members of the miR-135 family activate Wnt/β-catenin signaling through the inhibition of GSK3β expression.

## Discussion

It is widely accepted that glomerular visceral epithelial cells, also known as podocytes, are a critical target of injury in a wide variety of kidney diseases (31). The present study primarily demonstrated that miR-135a and miR-135b were significantly upregulated in the glomeruli of mice and in cultured podocytes treated with ADR, and that miR-135a and miR-135b are potential diagnostic biomarkers for glomerular and podocyte injury. A number of signaling pathways have been demonstrated to be involved in podocyte injury and apoptosis (32); however, the molecular mechanisms underlying podocyte injury remain poorly understood. The present study demonstrates that the ectopic expression of miR-135a and miR-135b promotes Wnt/β-catenin signaling and results in podocyte injury, which suggests that miR-135a and miR-135b are novel detrimental factors for podocytes and cause podocyte injury through the activation of Wnt/β-catenin signaling. Thus, we propose a novel mechanism underlying podocyte damage and suggest that podocyte injury is mediated by Wnt/β-catenin signaling.

Thus far, a great deal of understanding on podocyte biology, particularly in the regulation of the actin cytoskeleton, a main determinant of the complex architecture for podocyte function, that facilitates the identification of therapeutic targets for podocyte-associated diseases, has been achieved. For example, cyclosporine A (33) and rituximab (34) have been shown to protect podocytes from injury and dysfunction through the stabilization of the podocyte cytoskeleton. In the present study, we found that miR-135a and miR-135b are the detrimental factors for the stabilization of the podocyte cytoskeleton and that they disrupt the location of the actin skeleton. Thus, this suggests that the disruption of the cytoskeleton arrangement may be the main mechanism underlying podocyte dysfunction induced by miR-135a and miR-135b.

miRNAs are a class of short non-coding RNAs. In this study, using bioinformatics software, we found that GSK3β is a potential target gene of miR-135a and miR-135b. As is well known (35), GSK3β is an inhibitor of Wnt/β-catenin signaling and, thus, it was speculated that miR-135a and miR-135b may promote Wnt/β-catenin signaling through the suppression of GSK3β. Using luciferase reporter assays, western blot analysis and RT-qPCR, GSK3β was found to be a target gene of miR-135a and miR-135b. Adenomatous polyposis coli (APC), another inhibitor of Wnt/β catenin signaling, has been identified as a target of miR-135a and miR-135b in humans (13,14). However, the target sites of APC for miR-135a and miR-135b are not conserved between humans and mice. Therefore, we did not find that miR-135a and miR-135b inhibits APC expression in mice (data not shown), which is consistent with the results of the study by Nagel *et al* (13).

Despite a better understanding of the pathophysiological mechanisms of podocytes in kidney disease, only a few findings may be translated into more effective treatments due to difficulties in identifying functionally relevant podocyte-associated molecules. Hence, the need for the identification of the molecular mechanisms responsible for podocyte injury is imperative. The miR-135 family has 2 members, miR-135a and miR-135b. In the present study, we found that these 2 members were upregulated in models of podocyte injury and in glomeruli from patients with FSGS, indicating that there may be a common signal promoting high levels of miR-135a and miR-135b expression. The identification of this signal would decisively contribute to the development of novel drugs for the treatement of renal diseases associated with podocyte injury.

In conclusion, in the present study, we primarily found that miR-135a and miR-135b were upregulated in models of podocyte injury and in glomeruli from patients with FSGS. To the best of our knowledge, the present study is the first to demonstrate that miR-135a and miR-135b activate Wnt/β-catenin signaling through the inhibition of GSK3β in podocytes, suggesting a novel mechanism underlying miR-135a- and miR-135b-mediated podocyte injury. Our findings also suggests that miR-135a and miR-135b are potential diagnostic biomarkers for podocyte injury and that their inhibition may be a potential clinical therapeutic strategy for the treatment of podocytopathy. Although the findings of the current study demonstrate that miR-135a and miR-135b are upregulated by ADR in podocytes, the exact mechanisms through which ADR regulates miR-135a and miR-135b expression in podocytes remain unknown; thus, further research is required on this matter. In the future, our intention is to generate the ectopic expression and knockdown of these miRNAs in a mouse model in order to investigate the functions of miR-135a and miR-135b and the association between miR-135 family members and podocyte injury *in vivo*.

## Figures and Tables

**Table I:** Sequences of the primers used in RT-qPCR.

Name	GenBank accession no.	Sense	Antisense	Product size (bp)
Desmin	NM_010043	TGCAGCCACTCTAGCTCGTA	GACATGTCCATCTCCACCTG	150
Snail	NM_011427	AGCCCAACTATAGCGAGCTG	CCAGGAGAGAGTCCCAGATG	150
Nephrin	NM_019459	CGCCACCTGGTCGTAGATTC	ACCCCTCTATGATGAAGTAC	94
E-cadherin	NM_009864	GGCCAGTGCATCCTTCAAAT	CAGGTCTCCTCATGGCTTTG	148
WT1	NM_144783	CCGTGTGGTTCTCACTCTCA	CAAATGACCTCCCAGCTTGA	151
18s	NR_003278	GTAACCCGTTGAACCCCATT	CCATCCAATCGGTAGTAGCG	151

## References

[1] Nelson WJ, Nusse R (2004). Convergence of Wnt, beta-catenin, and cadherin pathways. Science.

[2] Teixeira VP, Blattner SM, Li M, Anders HJ, Cohen CD, Edenhofer I, Calvaresi N, Merkle M, Rastaldi MP, Kretzler M (2005). Functional consequences of integrin-linked kinase activation in podocyte damage. Kidney Int.

[3] Dai C, Stolz DB, Kiss LP, Monga SP, Holzman LB, Liu Y (2009). Wnt/beta-catenin signaling promotes podocyte dysfunction and albuminuria. J Am Soc Nephrol.

[4] Cai J, Schleidt S, Pelta-Heller J, Hutchings D, Cannarsa G, Iacovitti L (2013). BMP and TGF-β pathway mediators are critical upstream regulators of Wnt signaling during midbrain dopamine differentiation in human pluripotent stem cells. Dev Biol.

[5] Kato H, Gruenwald A, Suh JH, Miner JH, Barisoni-Thomas L, Taketo MM, Faul C, Millar SE, Holzman LB, Susztak K (2011). Wnt/β-catenin pathway in podocytes integrates cell adhesion, differentiation, and survival. J Biol Chem.

[6] Jackson RJ, Standart N (2007). How do microRNAs regulate gene expression?. Sci STKE.

[7] Long J, Wang Y, Wang W, Chang BH, Danesh FR (2010). Identification of microRNA-93 as a novel regulator of vascular endothelial growth factor in hyperglycemic conditions. J Biol Chem.

[8] Chen YQ, Wang XX, Yao XM, Zhang DL, Yang XF, Tian SF, Wang NS (2011). MicroRNA-195 promotes apoptosis in mouse podocytes via enhanced caspase activity driven by BCL2 insufficiency. Am J Nephrol.

[9] Wu J, Zheng C, Fan Y, Zeng C, Chen Z, Qin W, Zhang C, Zhang W, Wang X, Zhu X (2014). Downregulation of microRNA-30 facilitates podocyte injury and is prevented by glucocorticoids. J Am Soc Nephrol.

[10] Kato M, Zhang J, Wang M, Lanting L, Yuan H, Rossi JJ, Natarajan R (2007). MicroRNA-192 in diabetic kidney glomeruli and its function in TGF-beta-induced collagen expression via inhibition of E-box repressors. Proc Natl Acad Sci USA.

[11] Chung AC, Huang XR, Meng X, Lan HY (2010). miR-192 mediates TGF-beta/Smad3-driven renal fibrosis. J Am Soc Nephrol.

[12] Shi S, Yu L, Chiu C, Sun Y, Chen J, Khitrov G, Merkenschlager M, Holzman LB, Zhang W, Mundel P, Bottinger EP (2008). Podocyte-selective deletion of dicer induces proteinuria and glomerulosclerosis. J Am Soc Nephrol.

[13] Nagel R, le Sage C, Diosdado B, van der Waal M, Oude Vrielink JA, Bolijn A, Meijer GA, Agami R (2008). Regulation of the adenomatous polyposis coli gene by the miR-135 family in colorectal cancer. Cancer Res.

[14] Holleman A, Chung I, Olsen RR, Kwak B, Mizokami A, Saijo N, Parissenti A, Duan Z, Voest EE, Zetter BR (2011). miR-135a contributes to paclitaxel resistance in tumor cells both in vitro and in vivo. Oncogene.

[15] Zhou W, Li X, Liu F, Xiao Z, He M, Shen S, Liu S (2012). MiR-135a promotes growth and invasion of colorectal cancer via metastasis suppressor 1 in vitro. Acta Biochim Biophys Sin (Shanghai).

[16] Yamada Y, Hidaka H, Seki N, Yoshino H, Yamasaki T, Itesako T, Nakagawa M, Enokida H (2013). Tumor-suppressive microRNA-135a inhibits cancer cell proliferation by targeting the c-MYC oncogene in renal cell carcinoma. Cancer Sci.

[17] Wu S, Lin Y, Xu D, Chen J, Shu M, Zhou Y, Zhu W, Su X, Zhou Y, Qiu P, Yan G (2012). MiR-135a functions as a selective killer of malignant glioma. Oncogene.

[18] Anderegg A, Lin HP, Chen JA, Caronia-Brown G, Cherepanova N, Yun B, Joksimovic M, Rock J, Harfe BD, Johnson R, Awatramani R (2013). An Lmx1b-miR135a2 regulatory circuit modulates Wnt1/Wnt signaling and determines the size of the midbrain dopaminergic progenitor pool. PLoS Genet.

[19] He F, Peng F, Xia X, Zhao C, Luo Q, Guan W, Li Z, Yu X, Huang F (2014). MiR-135a promotes renal fibrosis in diabetic nephropathy by regulating TRPC1. Diabetologia.

[20] Fogo AB (2003). Animal models of FSGS: Lessons for pathogenesis and treatment. Semin Nephrol.

[21] Wang Y, Wang YP, Tay YC, Harris DC (2000). Progressive adriamycin nephropathy in mice: Sequence of histologic and immunohistochemical events. Kidney Int.

[22] Ni L, Saleem M, Mathieson PW (2012). Podocyte culture: Tricks of the trade. Nephrology (Carlton).

[23] Mundel P, Reiser J, Zúñiga Mejía Borja A, Pavenstädt H, Davidson GR, Kriz W, Zeller R (1997). Rearrangements of the cytoskeleton and cell contacts induce process formation during differentiation of conditionally immortalized mouse podocyte cell lines. Exp Cell Res.

[24] Kolfschoten IG, van Leeuwen B, Berns K, Mullenders J, Beijersbergen RL, Bernards R, Voorhoeve PM, Agami R (2005). A genetic screen identifies PITX1 as a suppressor of RAS activity and tumorigenicity. Cell.

[25] Bae GU, Lee JR, Kim BG, Han JW, Leem YE, Lee HJ, Ho SM, Hahn MJ, Kang JS (2010). Cdo interacts with APPL1 and activates Akt in myoblast differentiation. Mol Biol Cell.

[26] Bae GU, Kim BG, Lee HJ, Oh JE, Lee SJ, Zhang W, Krauss RS, Kang JS (2009). Cdo binds Abl to promote p38alpha/beta mitogen-activated protein kinase activity and myogenic differentiation. Mol Cell Biol.

[27] Pollak MR (2002). Inherited podocytopathies: FSGS and nephrotic syndrome from a genetic viewpoint. J Am Soc Nephrol.

[28] Wang Y, Jarad G, Tripathi P, Pan M, Cunningham J, Martin DR, Liapis H, Miner JH, Chen F (2010). Activation of NFAT signaling in podocytes causes glomerulosclerosis. J Am Soc Nephrol.

[29] Welsh GI, Saleem MA (2012). The podocyte cytoskeleton - key to a functioning glomerulus in health and disease. Nat Rev Nephrol.

[30] Wang D, Dai C, Li Y, Liu Y (2011). Canonical Wnt/β-catenin signaling mediates transforming growth factor-β1-driven podocyte injury and proteinuria. Kidney Int.

[31] Asanuma K (2015). The role of podocyte injury in chronic kidney disease. Nihon Rinsho Meneki Gakkai Kaishi.

[32] Kato H, Susztak K (2012). Repair problems in podocytes: Wnt, Notch, and glomerulosclerosis. Semin Nephrol.

[33] Faul C, Donnelly M, Merscher-Gomez S, Chang YH, Franz S, Delfgaauw J, Chang JM, Choi HY, Campbell KN, Kim K, Reiser J, Mundel P (2008). The actin cytoskeleton of kidney podocytes is a direct target of the antiproteinuric effect of cyclosporine A. Nat Med.

[34] Fornoni A, Sageshima J, Wei C, Merscher-Gomez S, Aguillon-Prada R, Jauregui AN, Li J, Mattiazzi A, Ciancio G, Chen L (2011). Rituximab targets podocytes in recurrent focal segmental glomerulosclerosis. Sci Translat Med.

[35] Nakamura T, Hamada F, Ishidate T, Anai K, Kawahara K, Toyoshima K, Akiyama T (1998). Axin, an inhibitor of the Wnt signalling pathway, interacts with beta-catenin, GSK-3beta and APC and reduces the beta-catenin level. Genes Cells.

